# Using a staircase procedure for the objective measurement of auditory stream integration and segregation thresholds

**DOI:** 10.3389/fpsyg.2013.00534

**Published:** 2013-08-20

**Authors:** Mona Spielmann, Erich Schröger, Sonja A. Kotz, Thomas Pechmann, Alexandra Bendixen

**Affiliations:** ^1^Department of Psychology, University of LeipzigLeipzig, Germany; ^2^Max Planck Institute for Human Cognitive and Brain SciencesLeipzig, Germany; ^3^School of Psychological Sciences, The University of ManchesterManchester, UK; ^4^Department of Linguistics, University of LeipzigLeipzig, Germany; ^5^Department of Psychology, Cluster of Excellence “Hearing4all”, European Medical School, Carl von Ossietzky University of OldenburgOldenburg, Germany

**Keywords:** auditory scene analysis, perceptual grouping, threshold measurement, psychophysics, adaptive method, streaming, auditory streams

## Abstract

Auditory scene analysis describes the ability to segregate relevant sounds out from the environment and to integrate them into a single sound stream using the characteristics of the sounds to determine whether or not they are related. This study aims to contrast task performances in objective threshold measurements of segregation and integration using identical stimuli, manipulating two variables known to influence streaming, inter-stimulus-interval (ISI) and frequency difference (Δf). For each measurement, one parameter (either ISI or Δf) was held constant while the other was altered in a staircase procedure. By using this paradigm, it is possible to test within-subject across multiple conditions, covering a wide Δf and ISI range in one testing session. The objective tasks were based on across-stream temporal judgments (facilitated by integration) and within-stream deviance detection (facilitated by segregation). Results show the objective integration task is well suited for combination with the staircase procedure, as it yields consistent threshold measurements for separate variations of ISI and Δf, as well as being significantly related to the subjective thresholds. The objective segregation task appears less suited to the staircase procedure. With the integration-based staircase paradigm, a comprehensive assessment of streaming thresholds can be obtained in a relatively short space of time. This permits efficient threshold measurements particularly in groups for which there is little prior knowledge on the relevant parameter space for streaming perception.

## Introduction

Every day our auditory system is confronted with a wide range of sounds, some of which may be more relevant to us than others such as the voice of a friend we are listening to at a noisy party. Our auditory system is able to focus on this particular sound information, separating it from all the other auditory objects or streams around us, such as other conversations, music, knives and forks clicking, or the street noise drifting up through the window. Additionally, the sounds belonging to one source can be integrated together into one stream, allowing us to hear a continuous sequence that is the story our friend is telling us rather than unconnected individual noises. This ability, which allows us performing such a complex organization of our auditory surroundings, is generally known as “Auditory Scene Analysis” (Bregman, 1990), and was initially described by the “Cocktail Party Effect” (Cherry, 1953). The auditory system segregates the relevant sounds from other, distracting sounds, which can then be integrated into an auditory stream.

In the laboratory, the phenomena of stream segregation and integration can be examined using two differing sounds, A and B. If these two sounds are alternated in time (ABBABBABB) they may be heard as integrated into one stream. However, under certain conditions, they may also seem to “split” or segregate, so that the listener hears two rather than one stream of sound. These two streams each correspond to the repetitions of one of the two sounds, here A--A--A-- accompanied by -BB-BB-BB (Van Noorden, 1975; Anstis and Saida, 1985).

Various factors can influence our ability to integrate or segregate two streams, but two factors that are known to have the greatest influence on streaming thresholds are inter-stimulus interval (ISI), and frequency separation (Δf) (Bregman et al., 2000). Generally, sequences with shorter ISI or larger Δf between the tones will segregate into separate streams more easily, whilst those with longer ISI or smaller Δf are more likely to be perceived as being integrated into one stream. Further, it is possible for people to control the ability to stream to a certain degree, allowing them to select whether to hear a tone sequence as either one or two streams, within certain parameters, through their attentional set (Van Noorden, 1975; Pressnitzer and Hupé, 2006).

Whilst the study of auditory streaming has spawned a large body of literature since it was first described (Ortmann, 1926; Miller and Heise, 1950; Bregman and Campbell, 1971; Van Noorden, 1975), psychoacoustic measurement of streaming has in recent years experienced a resurgence in popularity in psychological, as well as in animal behaviorist and neuroscientific research of this phenomenon (Carlyon, 2004; Carlyon and Gockel, 2007; Bee and Micheyl, 2008; Ahveninen et al., 2011). Furthermore, streaming paradigms are increasingly being used to research other aspects of auditory perception, which may be dependent on object formation, such as comodulation masking release (Dau et al., 2003, 2009).

There are a variety of approaches by which auditory stream segregation and integration can be measured, both with and without asking for an explicit subjective report from the participants. When measuring streaming thresholds subjectively, subjects are usually asked explicitly whether they perceive the tones as one or two streams. The advantage of this type of measure is that it is easy to set up, and a direct report of the subject's perception of the tones can be recorded. However, the disadvantage is that this type of measurement cannot be used in a paradigm in which subjects are not attending to the stimuli as can be the case in electroencephalography (EEG) studies. Further, some subjects, especially children or clinical populations, may find it difficult to give a good subjective report on whether or not they perceive one or two streams. This difficulty may be exacerbated by tasks, which are often performed in a quite unnatural situation, and by the fact that subjects may feel a pressure to “do well.”

By asking subjects to complete a perceptual task that is supported either by an integrated or a segregated percept, it becomes possible to measure auditory streaming thresholds objectively, i.e., without explicitly asking about the subject's perception of the streams. For example, it is easier to detect particular details about one stream, such as its regularities and any deviant contained within it, if it is not integrated with the second stream. The second stream may hide the regularities of the first, thus making deviants harder to detect (Sussman et al., 2001, 2007a; Winkler et al., 2003a,b; Sussman and Steinschneider, 2006, 2009). In contrast, the temporal relationship between the tones of two streams is easier to judge when they are integrated into one stream (Bregman and Campbell, 1971; Vliegen et al., 1999; McAnally et al., 2004). When the cohesion between the higher and lower tones is lost and streaming occurs, differences in the ISI between the higher and lower tones become very difficult to detect. Apart from being of use in objective behavioral studies, where attention is focussed on the task and an overt response must be given, such objective measures of integration and segregation can also be used in studies examining the role of attention (Sussman and Steinschneider, 2009), in neuroscientific studies to examine neural correlates of streaming (Micheyl et al., 2007; Snyder and Alain, 2007; Sussman et al., 2007a), as well as in infant and animal studies (Demany, 1982; Winkler et al., 2003a; Fay, 2007), as they can be taken without requiring the attention of the subject by using imaging or EEG methods, where no explicit behavioral response is required from the subject. Instead, brain responses that are assumed to be linked to the detection of deviants are recorded. For example, in EEG studies, the elicitation of the MMN component has often been considered to reflect the physiological detection of deviants in both attended and unattended listening paradigms (Novak et al., 1990).

Looking at the approaches described and used in different studies, it becomes clear that there is a certain lack of uniformity when it comes to how streaming is measured. This makes it difficult to compare the outcome of different studies directly. Different stimuli, different paradigms, and different measurement methods in different laboratories, all collected using different subjects, would make any comparison attempted very difficult. Under these circumstances, it is important to examine some of these approaches in a systematic fashion, using the same subjects and stimuli, with data recorded in the same experiment under very similar conditions. This would permit a direct comparison of integration and segregation thresholds, measured both subjectively and objectively. There is, as far as we are aware, only one study that looked to perform a systematic comparison of tasks for the measurement of stream integration and segregation (Micheyl and Oxenham, 2010). In this study, Micheyl and Oxenham measured task performance at particular combinations of ISI and Δf values. However, this previous study sampled the parameter space quite sparsely with a small group of 7 subjects, who took part in 2 h testing sessions 2–3 times per week for the duration of the study. As responses were taken at 2 different ISI levels and 6 different Δf levels, this would give data for only 12 different points for each of the objective tasks. However, when working with special groups such as children or clinical groups, the parameter space required for the study can be a lot more variable and less simple to predict, and it may be difficult to pre-select at which ISI or Δf levels to test. Based on the amount of time that the Micheyl and Oxenham study was reported to take, to extend such an approach to allow for the coverage of an extended parameter space of the variables involved would take much too long. We would therefore suggest that it is necessary to look at other means of threshold determination that require fewer limitations on the parameter space.

One reason for the long duration of the measurement is that in most studies, fixed combinations of ISI and Δf values are determined in advance. Measurements must then be taken at each of these fixed points for each subject, regardless of whether the particular ISI-Δf combination is close to their streaming threshold or not. This may lead to large amounts of time spent measuring ceiling or floor effects. We propose to overcome this problem by setting only one parameter (ISI or Δf) as fixed, while varying the other one according to a staircase protocol. This would render the measurement considerably more time-efficient, allowing the coverage of a larger parameter space. Note that even though staircase procedures have been used in streaming studies before (Cusack and Roberts, 2000; Roberts et al., 2002, 2008; Micheyl and Oxenham, 2010), typically the staircase variation referred to secondary parameters measuring task performance, rather than to primary parameters directly affecting the streaming percept (e.g., ISI or Δf). By secondary parameters, we refer to variables that affect the difficulty of the perceptual task used to assess the streaming percept, such as the amount of an across-stream temporal shift that participants are instructed to detect in order to measure their success in integrating two streams. In contrast, primary parameters are those that have a direct effect on whether one or two streams are perceived. By manipulating secondary parameters, one can determine the streaming percept or the ease with which this percept can be held. One can, however, not directly assess the streaming threshold, that is, the point where the percept switches from integrated to segregated and vice versa. Measuring streaming thresholds based on secondary parameters would require multiple runs of the staircase protocol at different, pre-set combinations of primary task parameters, and possibly interpolation between these pre-set values. In contrast, manipulating primary task parameters in a manner adapted to the current task performance of the participant (e.g., with a staircase procedure) yields a direct measurement of the streaming threshold in terms of a combination of ISI and Δf values for which perception is exactly at the switching point between segregation and integration.

A staircase procedure for directly targeting the streaming threshold has been used in some studies (McAnally et al., 2004), yet these examined just one streaming parameter, and only tested thresholds for one type of streaming percept (integration). To our knowledge, a systematic attempt to test the two most important streaming parameters (ISI and Δf) and to compare streaming thresholds for integration and segregation in a staircase procedure has not been undertaken. This would therefore be a first attempt to see if it would be possible to use a staircase method to explore both stream integration and segregation without a clearly predefined parameter space.

By using both ISI and Δf as variables in a staircase procedure, the current study attempts to find a paradigm that will give a good coverage of the parameter space to be tested by removing the necessity of pre-selecting a certain number of fixed values at which to measure. Another aim of the study is also to complete all threshold measurements to be compared in one 75–90 min session for each subject by exploiting the efficiency of the staircase measurement procedure, thereby allowing within-subjects comparisons without the burden of multiple testing sessions.

Two objective tasks were chosen for the current study, one that is supported by hearing two segregated streams, here called the intensity task, and one that is supported by hearing one integrated stream, here called the rhythm task. The intensity task is based on a paradigm used by Sussman et al. (2007b, see also Sussman and Steinschneider, 2009) in studies of childhood stream segregation, as the task should be generalizable for use with children and possibly other participants who may have difficulties with more complex instructions. For the same reasons, the rhythm task is based on a paradigm used by McAnally et al. in a study with dyslexic children (2004). Δf and ISI fixed values were borrowed in part from previous studies (Helenius et al., 1999; McAnally et al., 2004; Sussman et al., 2007b; Sussman and Steinschneider, 2009; Micheyl and Oxenham, 2010), but were partly also expanded to provide a greater range. This was done to make the task transferable to children as well, who have been suggested to have higher stream segregation thresholds than adults (Sussman and Steinschneider, 2009). A subjective measure of the streaming percept held was taken as well, making it possible to compare and examine how manipulating key parameters affects perceptual organization, both subjectively and objectively. Again, we chose a task that had been used with an atypical population, this time dyslexic adults (Helenius et al., 1999), to try to ensure that the task would be more widely transferable. As there is a range within the parameter space measured where it is possible to actively influence whether a percept is heard as segregated or not (Van Noorden, 1975; Pressnitzer and Hupé, 2006), we feel it is important to look at methods that would allow the identification of thresholds where a segregated percept becomes difficult to hold, as well as those where an integrated percept becomes difficult to hold. Using these tasks, we looked to identify segregation and integration thresholds based on ISI and Δf, so that we might be able to provide a more comprehensive investigation of auditory streaming boundaries.

## Experiment 1

### Subjects

Twenty healthy adult subjects participated in the first experiment. Of these, 19 subjects (3 male, 16 female; mean age 23.63, *SD* = 6.26) were analyzed, as one subject was unable to solve one of the training tasks after 8 attempts. All subjects provided written informed consent and had reportedly normal hearing. None of the subjects spoke any tonal languages or had received more than 4 years of musical schooling. None of the subjects with musical training had been musically active in the last 4 years. Subjects received credit points for their participation.

### Materials and methods

The experiment was set up to map out the influence of ISI and Δf on auditory stream segregation by looking at the stream segregation and integration thresholds. For the objective measures, this was done using a weighted 3 up—1 down staircase procedure (Kaernbach, 1991). Short (2.6–6 s, depending on ISI) sound sequences were presented, after which subjects had to give a response by pressing one of two buttons on a response keypad, using their left and right thumbs. If they gave a correct answer, suggesting that they were still able to hear the percept that facilitated the task, the difficulty was increased by one step, whilst an incorrect response led to the difficulty being reduced by three steps. The next sequence was only triggered once the subject had given a response. Subjects completed twelve objective measurement blocks that were presented in a pseudo-randomized order, with the four blocks belonging to each of the three objective tasks being grouped. In addition, eight subjective threshold measurements were taken using a self-adjustment procedure, giving a total of twenty blocks. Before each set of four objective measurement blocks, subjects completed a training block, which was repeated until the subjects declared themselves to be confident in having understood the task. The tasks presented were as detailed below.

**Figure 1:**
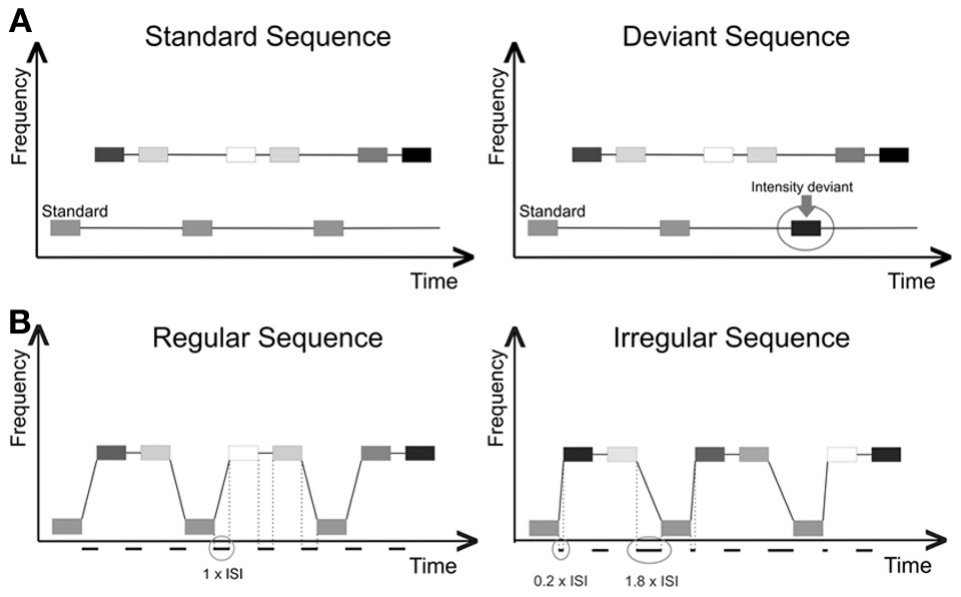
**(A)** Intensity task set up: pure tones with 50 ms duration were presented at differing intensity levels (darker shading indicates higher intensity). The high-stream tones were presented with a random level of 65, 75, or 85 dB SPL. The low-stream tones (250 Hz) were played with a level of 70 dB SPL (standards). Half of the sequences contained one low-stream tone with a level of 80 dB SPL (deviant). Subjects were asked to identify whether or not the sequence contained a deviant tone. **(B)** Rhythm task set up: pure tones with 50 ms duration were presented at differing intensity levels (darker shading indicates higher intensity). The high-stream tones were presented with a random level of 65, 75, or 85 dB SPL. The low-stream tones (250 Hz) were played with a level of 70 dB SPL. In each trial, the subject was played a sequence that would need to be identified as either a regular sequence (all ISI are equal in length) or an irregular sequence (the ISI between the low tone and the first high tone is 80% shorter than the ISI in the regular sequence). To retain the same time difference between the low stream tones, the gap between the second high stream tone and the next low stream tone in a deviant sequence was lengthened.

1. Objective threshold measurement, intensity task (easier in two-stream percept), pre-determined ISI levels, variable Δf. Pure sinusoidal tones of 50 ms duration were presented over Sennheiser HD25-1 closed back on-ear headphones. A sequence with a regular ABB pattern consisting of 3 tones (one fixed lower tone at 250 Hz and with a level of 70 dB SPL, and two variable higher tones with variable frequencies and random levels of 65, 75, or 85 dB SPL) was used to determine the Δf threshold. All sound levels were measured using a HEAD acoustics HMS III.0 artificial head measurement system. The overall length of each sequence was 25 tones (i.e., ending in … ABBABBA). The sequences were presented at a fixed ISI pre-set at one of four values (50, 100, 150, or 200 ms, manipulated block-wise). We decided to use values 50 ms apart as this was the difference between the two ISIs at which Micheyl and Oxenham (2010) had measured, and appeared to be effective at showing a difference in the stream perception. Within each block, Δf was varied from sequence to sequence, with the starting point being set at 15 semitones (ST) difference. A deviant tone was introduced in 50% of the sequences, where a pseudo-randomly chosen low tone was presented at a louder intensity than the other low tones (80 dB SPL, see Figure 1A).
  The deviant occurred by random choice in position 5, 6, 7, or 8 of the low-tone sequence (i.e., not in the initial positions 1–4, and not in the final position 9). The subjects were asked to press a button during or after each sequence to indicate whether or not they heard a louder deviant tone in the lower sound sequence. In order to make efficient use of time, trials were triggered by the subject's button press, rather than after a set amount of time. The Δf decreased by 1 ST if the subject answered correctly, or increased by 3 ST if the answer was incorrect. As it should be more difficult to identify infrequent within-stream deviants if all tones are perceived as belonging to one stream (Winkler et al., 2003b), subjects should only be able to reliably identify whether or not the louder deviant was present in the lower sequence when the sounds can still be perceived as two segregated streams. The run was terminated after 14 reversals, and the average of the Δf values at the last eight reversals was taken as an estimate of the threshold.
2. Objective threshold measurement, rhythm task (easier in one-stream percept), pre-determined ISI levels, variable Δf. Subjects were asked to listen to sequences made up of the same pattern of pure tones as in the previous task (low-high-high). Sequences for this task were only 12 tones long (4 ABB patterns) as this was found sufficient for rhythm discrimination in a pilot study. Intensity variations from task 1, whilst not relevant to this task, were preserved to make the stimuli more comparable. In this case though, the subjects needed to identify whether the entire sequence of high and low tones had an overall regular ISI pattern. The subject was presented with a sequence, which could either be a regular sequence, in which the tones were all separated by an ISI of equal length, or an irregular sequence, in which the ISI between the low A tone and the first higher B tone was reduced by 80%, whilst the ISI between the 2nd B tone and the next A tone was prolonged accordingly, in order to maintain the same overall pace as in the regular sequences (see Figure 1B).
  The probability of an irregular sequence being presented was 50%. The subjects were asked to press a button during or after each sequence to indicate whether they had heard a regular or an irregular sequence. The sequences were presented over 4 blocks, with an ISI of 50, 100, 150, or 200 ms. The starting Δf in each block was 2 ST. Results were obtained using a 1-up 3-down staircase procedure, in which Δf increased by 1 ST if the subject answered correctly or decreased by 3 ST if they answered incorrectly. As it should be more difficult to perceive the relationship between the low and high tones when they are segregated, subjects should only be able to reliably determine whether the sequence had a regular or irregular rhythm when the sequence can still be perceived as one integrated stream (Bregman and Campbell, 1971). Again, for efficiency trials were triggered by the subject's button press, rather than after a set amount of time. The run was terminated after 14 reversals, and the average of the Δf values at the last eight reversals was taken as an estimate of the threshold.
3. Objective threshold measurement, rhythm task (easier in one-stream percept), pre-determined Δf levels, variable ISI. Subjects performed the rhythm task as described in task 2. However, this time the ISI was variable and the Δf was fixed. Four blocks were presented with a Δf of 10, 17, 24, and 31 ST, respectively. The starting ISI in each block was set at 200 ms. Subjects were asked to press a button during or after each sequence to indicate whether they had heard a regular or an irregular sequence. Results were obtained using a 1-up 3-down staircase procedure, in which the ISI decreased by 10 ms if the subject answered correctly or increased by 30 ms if they answered incorrectly. The 10 ms step size was chosen as this was the smallest unit used by Helenius et al. (1999) in their self-adjustment study. As the self-adjustment part of the experiment was based on that done by Helenius, and as it should be as comparable as possible to the objective measurement, this step size was also implemented in the objective measurement.
  The first 5 subjects in the study also performed an objective ISI threshold measurement at 4 fixed Δf values for the intensity task to get an ISI threshold for the segregated percept, but these measurements were not continued, as all subjects performed at ceiling and were able to correctly identify the intensity deviant in close to 100% of responses. Even though longer ISIs are known to impede segregation, performance actually improved as the ISI became longer. This was probably because as the ISI became very long, the task became easy to solve cognitively without having to rely on being able to segregate the streams (i.e., by focusing on the intensity of every third tone; see Dowling et al., 1987).
4. Subjective threshold adjustment, pre-determined ISI levels, variable Δf. Subjects were asked to listen to a continuous sound sequence made up of the same pattern of tones as in the previous tasks. By means of the two response buttons, they were able to adjust the Δf between the tones themselves until they felt they could consistently only hear a two-stream percept. Note that this instruction implies that an integration threshold (i.e., the point at which integration is no longer possible) is measured, rather than a segregation threshold. Subjects completed one self-adjustment task for each of the fixed ISI lengths (50, 100, 150, 200 ms).
5. Subjective threshold adjustment, pre-determined Δf, variable ISI. Subjects were asked to listen to a continuous sound sequence made up of the same pattern of tones as in the previous tasks. By means of the two response buttons, they were able to adjust the ISI between the tones themselves until they felt they could consistently only hear a two-stream percept. Again, it should be noted that this instruction implies that the integration rather than the segregation threshold is measured. Subjects completed one self-adjustment task for each of the fixed Δf values (10, 17, 24, and 31 ST).

We would expect subjects to show an increase in the Δf required for them to perceive the tones as two segregated streams as the ISI increases (task 1). We would also expect an increase in the Δf up to which subjects are able to hold an integrated percept as the ISI increases (task 2), and an increase in the ISI up to which subjects are able to hold an integrated percept as the Δf increases (task 3). Further, we would expect there to be a difference between the thresholds measured for integration and those measured for segregation, as shown by Van Noorden (1975). Finally, we would expect the subjective thresholds (self-adjustment tasks 4 and 5) to correlate with the objectively measured thresholds for integration. This would help validate the objective tasks as providing a good measure of the perceptual threshold.

### Results

Kolmogorov–Smirnov tests show that data in tasks 1 (100, 150, and 200 ms conditions), 2 (50 and 100 ms conditions), 3 (10ST condition), and 4 (50 ms condition) has a non-Gaussian distribution with a positive skew (all *p* < 0.05). We therefore performed a log transformation on the data of all conditions to reduce the skew. All further analyses are based on the log-transformed values. For each of the five tasks, a repeated measures analysis of variance (ANOVA) was calculated to find out if there were significant differences between the thresholds measured at the four different ISI or Δf values. Significant ANOVA effects were examined further through trend analyses to determine if there was a proportional change in the dependent variable across the fixed values.

For the intensity task (task 1), which should require within-stream deviance detection supported by a segregated percept, an increase in the Δf thresholds with increasing ISI was expected, as subjects should find it more difficult to maintain a segregated percept at lower Δf and slower ISI. This would imply that a greater Δf would be necessary to hear the tones as two separate streams. However, contrary to this hypothesis, no significant difference between the Δf thresholds measured at the different ISI values was found [*F*_(3, 54)_ = 0.56, *p* = 0.65].

For the two rhythm tasks (tasks 2 and 3), which should require an across-stream temporal judgment supported by an integrated one-stream percept, an increase in the Δf thresholds with increasing ISI was expected, as subjects should find it easier to maintain an integrated percept at lower Δf and slower ISI. The ANOVAs show that the Δf threshold was affected significantly by ISI length (task 2) [*F*_(2.08, 37.50)_ = 47.65, *p* < 0.001, ε_GG_ = 0.694], and that ISI thresholds were also affected significantly by Δf (task 3) [*F*_(3, 54)_ = 29.9, *p* < 0.001]. Follow-up analyses reveal a linear trend for task 2, with the Δf measured increasing with longer ISI values [*F*_(1, 18)_ = 73.12, *p* < 0.001]. A linear trend was also found for task 3, with the ISI threshold generally increasing with larger Δf values [*F*_(1, 18)_ = 57.51, *p* < 0.001].

The results from the rhythm task with variable Δf (task 2) were checked against the rhythm task with variable ISI (task 3). For both tasks, task performance was expected to improve with longer ISIs or higher Δf values. As they are both measured with the same task within the same parameter space and participants, the resulting thresholds should also fall on the same curve when plotted. In view of the linear trend found for both tasks, this curve would best be approximated by assuming the same linear relation between Δf and ISI for both tasks. This was tested by calculating the correlation between Δf and ISI for each subject using the data from both tasks (8 pairs of values per subject). The resulting 19 correlation coefficients were *z*-transformed, and the mean *z*-score value was calculated and tested against zero [*z* = 1.43, *SE* = 0.45, *p* < 0.005]. The resulting mean *z*-score was then transformed back into a mean correlation coefficient [*r* = 0.89]. This shows that the results from the two tasks correspond well with each other.

**Figure 2:**
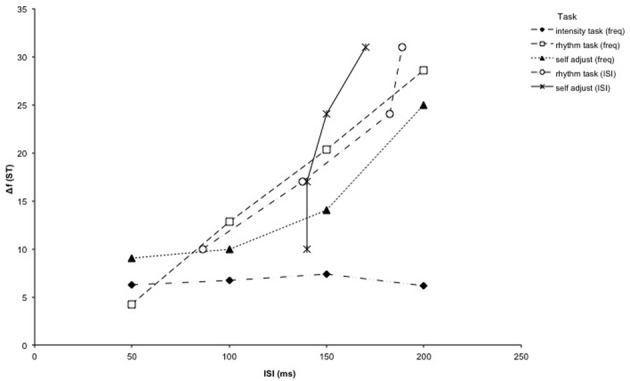
**Results of Experiment 1: The results for all objective tasks of Experiment 1 (one intensity deviance detection and two rhythm decision tasks) are plotted together, along with two subjective self-adjustment tasks (variable Δf or variable ISI, respectively)**. Tasks where Δf was varied are plotted with the Δf threshold as a function of the ISI length. For the variable ISI rhythm and self-adjustment tasks, the ISI threshold was plotted as a function of the Δf.

For the self-adjustment tasks, the ANOVA showed that the Δf thresholds were significantly affected by ISI length (task 4) [*F*_(3, 54)_ = 17.67, *p* < 0.001]. Follow-up analyses for task 4 reveal a linear trend for task 4 [*F*_(1, 18)_ = 45.38, *p* < 0.001]. The subjective thresholds measured in the variable ISI condition (task 5), however, did not follow the same pattern as the other integration threshold measurements (see Figure 2). ISI thresholds were not significantly affected by Δf (task 5) [*F*_(3, 54)_ = 1.29, *p* = 0.287].

The self-adjustments in task 4 were always done from an integrated one-stream percept, and subjects were asked to adjust the Δf until they could no longer hear the one-stream percept. This makes the results subjective measures of integration thresholds. To validate the objective measurement as a good predictor of the subjective perception of integration thresholds, the results of the subjective threshold measurement with variable Δf (task 4) can be compared with the objective results of the equivalent rhythm task (task 2).

The correlation between Δf and ISI was calculated for each subject using the data from both tasks (8 pairs of values per subject). The same method was used as above where task 2 and 3 were compared. The mean *r* was again significantly different from zero [*z* = 0.91, *SE* = 0.45, *r* = 0.72, *p* < 0.05]. Therefore, it can be assumed that the results from task 2 and 4 correspond to one another.

The results of the subjective threshold measurement with variable ISI (task 5) and the objective results of the equivalent rhythm task (task 3) were also compared using the same method. The mean *r* was not significantly different from zero in this case [*z* = 0.57, *SE* = 0.45, *r* = 0.51, *p* = 0.20]. The results of the two tasks can therefore be assumed to be uncorrelated, which is in line with the lack of increase in the ISI thresholds with increasing Δf that was observed in task 5.

### Discussion

If we assume that the rhythm task (tasks 2 and 3) can only be solved if subjects maintain an integrated percept, then, as expected, the results show that subjects were able to maintain a one-stream percept at higher Δf values when ISI lengths were longer. They were also able to maintain a one-stream percept at shorter ISI lengths when the Δf between the high and low tones was smaller. There was a significant correlation between the ISI length and the Δf up to which subjects were able to successfully solve the task (task 2), and these objective threshold measurements also correlated with the subjective thresholds reported by the subjects (task 4). Further, the thresholds measured in the rhythm task with variable Δf values (task 2) and those measured in the rhythm task with variable ISI values (task 3) were consistent, lending strong support to the assumption that the rhythm task threshold measurements do accurately reflect the perceptual integration thresholds of the subjects.

The results from the subjective variable ISI condition (task 5), however, do not fit the same pattern as the other integration threshold measurements. No significant variation could be found between the ISI thresholds for the four fixed Δf values that were measured. As all the other results from the integration threshold measurements show a similar pattern to each other, it would seem unlikely that the thresholds in task 5 should be invariable. It seems that varying the ISI at a constant Δf value for subjective perceptual judgments is not symmetric to varying the Δf at a constant ISI value. The reasons for this asymmetry remain to be explored. It is possible that the frequency separation change was more salient to the subjects than the ISI change, allowing subjects to better judge where their integration threshold lies. If it is more difficult for some subjects to subjectively determine at what point integration is no longer possible when ISI is varied and Δf is fixed, this may lead them to hold on to either a very stable or a highest possible threshold of integration, thereby obscuring differences between the different Δf levels. As long as the underlying reasons for these difficulties are unclear, we would suggest that subjective self-adjustment procedures of ISI stream segregation and integration thresholds should be approached and interpreted with caution.

Against the initial expectations, there was no significant difference in performance across the different ISI levels in the objective thresholds measured through the intensity task. The question therefore arises as to why the Δf thresholds do not show the expected increase with ISI in the current measurements in this task.

It could be suggested that there was a ceiling effect in this particular task, in the sense that streaming was still “too easy” at the ISIs measured in the current study. Although early reports would support this, suggesting that the employed ISI range of 50–200 ms may not lead to a large increase in Δf thresholds required for streaming (Van Noorden, 1975), some more recent studies which have found effects of ISI on the Δf needed to segregate streams at ISI values within the range used in the present study suggest otherwise (Denham et al., 2010, 2013; Micheyl and Oxenham, 2010).

Another possible explanation might be found in the suggestion that the segregated percept requires a certain build-up time. The build-up of streaming is usually associated with studies where sound streams were presented over 10 s or more (Carlyon et al., 2001), with the initial build-up lasting about 5 s, though it has more recently been suggested that segregation can be built up with a 2 s inducer sequence (Roberts et al., 2008). It may therefore also be argued that the sequences presented here were too short in the shorter ISI conditions for streaming to build up (2.4 s for the shortest ISI value), and that subjects were therefore less able to detect the deviant, especially if it occurred earlier in the tone sequence (depending on ISI, the first possible occurrence of the deviant is within 1.2–3 s). This might then explain a weaker performance in the task in the shorter ISI conditions, in spite of the fact that the shorter ISI would support stream segregation (Van Noorden, 1975), leading to the overall performance not showing any significant change across all four ISI conditions. However, other studies have shown the effect of build-up to be faster in shorter sequences when subjects were aware of the length of the sequence or where sequences of the same length were blocked together. This could be due to a certain level of readiness and increased attention, as the subjects knew in advance that they would not have long to listen to the tones presented (Pressnitzer, 2008). Under the present task instructions, where fast stream segregation was beneficial, it is possible that subjects were able to access a segregated percept almost immediately.

To see whether the current results had been affected by deviants being missed in faster sequences, as might be expected if build-up took longer than the time between the onset of the sound stream and the first deviant, a two-factorial repeated measures ANOVA was performed examining the effect of the deviant position within the tone sequence (4 levels: position 5/6/7/8) and of the ISI length (4 levels: 50/100/150/200 ms) on the percentage of correct responses for sequences containing a deviant. However, no significant effect of deviant position was found [*F*_(3, 54)_ = 2.131, *p* = 0.107]. Further, no significant effect of ISI length on percentage of correct responses was found either [*F*_(3, 54)_ = 1.325, *p* = 0.276], nor was there a significant interaction between ISI and deviant position [*F*_(9, 162)_ = 0.748, *p* = 0.665]. Therefore, as later deviants are not significantly easier to detect than early deviants, and deviants in slower sequences are not significantly easier to detect than in faster sequences, we would suggest that the current results cannot be attributed to subjects being unable to build up a segregated percept fast enough.

A third explanation might be that subjects were able to solve the task even when stream segregation was no longer possible. By employing some other strategy, such as tuning into the regular pace of the stimulus configuration and specifically attending every third (task-relevant) tone while ignoring the two intermediate tones (Dowling et al., 1987), subjects may have been able to determine if a deviant was present without a segregated percept.

A further explanation might be that as each sequence was triggered by the subject's response to the previous sequence consecutive sequences may have followed each other so closely that subjects were able to transfer their previous percept to the next sequence. Stream segregation has been shown to be cumulative (Bregman, 1990; Anstis and Saida, 1985), with longer sequences having a greater tendency to segregate, though these build up effects decay when there is a noticeable interruption to the sequence (Bregman, 1990; Rogers and Bregman, 1998; Haywood and Roberts, 2010). In studies of build-up effects on stream segregation, an inducer sequence consisting of one of the tone streams of the later test sequences was sufficient to strongly promote a segregated percept (Roberts et al., 2008). As the frequency of the lower tones, to which the subject needed to attend, remained the same, and as it is possible that subjects responded quickly enough that the gap between the two sequences was minimal, it may be that there was not sufficient disruption to the sequence to avoid a build-up of stream segregation across multiple sequences.

This would also be supported by studies of facilitation or priming effects on auditory stream perception: Some studies have revealed that the auditory system tends to retain a previously dominant percept in spite of parameter changes (Sussman and Steinschneider, 2006; Rahne and Sussman, 2009; Snyder et al., 2009). According to Rahne and Sussman (2009), this tendency is asymmetric, with segregation having a longer-lasting effect than integration on subsequent perceptual organization. It might therefore be that the intensity task was particularly susceptible to a transfer effect. This might explain subjects performing equally across all ISI levels, and therefore also the failure to find an effect of ISI on Δf threshold levels when measured using the intensity task. Though the results of Experiment 1 showed that there was no significant effect of deviant position, we cannot rule out an overall transfer effect across trials possibly facilitating stream segregation.

In order to reduce the likelihood of any possible ceiling effect, to counteract facilitation effects of perceptual organization in the previous sequence, and to make it more difficult to apply alternative strategies based on temporal attention, a follow-up experiment focussing on the intensity task (task 1) was conducted.

## Experiment 2

### Subjects

Ten healthy adult subjects participated in the second experiment (4 males, 6 females, mean age 22.3, *SD* = 1.49). All subjects provided written informed consent and had reportedly normal hearing. None of the subjects spoke any tonal languages or had more than 4 years of musical schooling, and none of the subjects had participated in Experiment 1. Subjects either received course credit points or payment for their participation.

### Materials and methods

This second experiment was set up to test the alternative explanations for the lack of change in the Δf threshold with increasing ISI during the intensity task in Experiment 1. All conditions employed an objective threshold measurement by means of the intensity task described in task 1 of Experiment 1, with pre-determined ISI levels and variable Δf. The subjects were asked to press one of two buttons on the response keypad with their left and right thumbs to indicate whether or not they heard a louder deviant tone in the lower sound sequence. This could be done either during or after each sequence. However, two manipulations were made to the stimulus setup from Experiment 1, a timing jitter and a base frequency jitter. The four resulting conditions for the study were therefore as follows:

1. Control. Stimuli and task were identical to those from task 1 of Experiment 1.
2. Jittered timing. Stimuli were the same as in the control condition, with the exception that the ISI was not kept constant between tones. Instead, the onset of each individual tone was independently jittered by a random amount that ranged equiprobably from −40 to +40% of the nominal ISI value.
3. Jittered frequency. Stimuli were the same as in the control condition, with the exception that a different frequency was pseudo-randomly chosen for the lower sequence for each trial. The maximum jitter was set to 300 Hz ± 5 ST. The difference between the lower tone sequence frequencies of two consecutive trials was at least 1 ST.
4. Jittered timing and frequency. Stimuli were the same as in the control condition, with the exception that both the frequency of the lower tone sequence was pseudo-randomly chosen for each sequence as in the jittered frequency condition (condition 3), and the ISI was randomly jittered as in the jittered timing condition (condition 2).

Firstly, by jittering the timing of stimulus presentation within each sequence, subjects should be prevented from being able to predict through the regularity of the sequence when the lower tones were being presented and just focussing on these. This manipulation has been shown to impair task performance in similar protocols (Andreou et al., 2011; Rimmele et al., 2012).

Secondly, by changing the frequency of the lower tones with every sequence, it should be more difficult for subjects to retain the previously experienced segregated percept by just “holding on” to the lower stream. This manipulation should make it more likely that participants will segregate the streams anew with each presented sequence.

Both these manipulations should make it more difficult to apply alternative cognitive strategies to solve the task. As the cognitive strategies become less reliable and the likelihood of a performance limit having been reached (i.e., a floor effect) is reduced, we would expect performance to become more influenced by stream segregation. If this is the case, it should be possible to see an influence of ISI length on the Δf threshold, with longer ISI tone sequences requiring a higher Δf to segregate the streams than shorter ISI tone sequences.

If, in spite of the manipulations to the stimuli, no difference to the thresholds can be found, then it would appear more likely that other factors are influencing the performance in the intensity deviant detection task apart from those actively being manipulated, and that we would have to look at aspects beyond those that usually influence stream segregation to determine why the thresholds remained so stable in the intensity task.

### Results

Kolmogorov–Smirnov tests showed that the data in 2 ISI levels of condition 2 (150 and 200 ms conditions) had a non-Gaussian distribution with a positive skew (*p* < 0.05). We therefore performed a log transformation on the data of all conditions to reduce the skew. All statistical analyses were performed on log-transformed data. Thresholds from the control condition were compared to those of the intensity task of Experiment 1 (task 1) by means of an independent-sample, two-tailed Student's *t* test. This was done to ensure that the subjects in Experiment 2 were not performing significantly differently to those who had taken part in Experiment 1. There was no significant difference between the two groups at any of the four ISI levels [50 ms: *t*_(27)_ = −0.30; 100 ms: *t*_(27)_ = 0.438; 150 ms: *t*_(27)_ = −0.54; 200 ms: *t*_(27)_ = −0.58], with all *p* > 0.5. This shows that the thresholds measured in the intensity task of Experiment 1 and in the control condition of the current experiment did not differ significantly, and that the subject performance should be comparable in both experiments.

**Figure 3:**
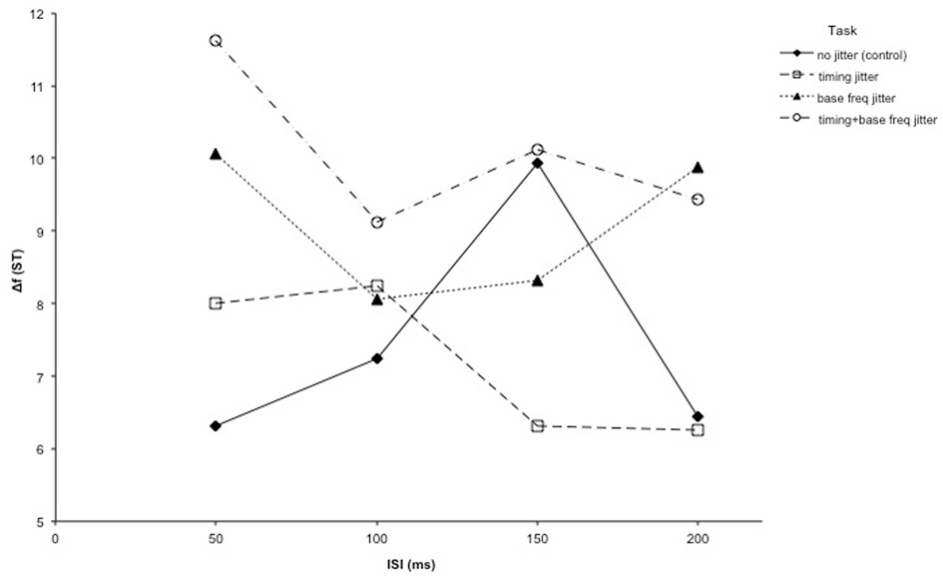
**Results of Experiment 2: Four variations of the intensity deviant detection task from Experiment 1 were performed in Experiment 2: an unaltered condition (control), a condition where each tone onset was jittered (timing), a condition where the frequency of the lower tones was jittered between trials (base freq), and one condition combining both jitter adjustments (timing + base freq)**. The thresholds for all 4 conditions are plotted together, with the Δf threshold shown as a function of the ISI length.

In order to be able to examine the effect of jittering the timing and the base frequency as well as the effect of ISI length, a 3-factorial ANOVA with the factors ISI (4 levels: 50/100/150/200 ms), timing jitter (2 levels: absent/present), and base frequency jitter (2 levels: absent/present) was performed. The results show there was a main effect of the base frequency being jittered [*F*_(1, 9)_ = 10.79, *p* < 0.01], with higher Δf thresholds observed for conditions with base frequency jitter (conditions 3 and 4) than for conditions without base frequency jitter (conditions 1 and 2). This suggests that jittering the base frequency did in fact cause subjects to be worse at solving the task. However, there was no significant effect of the timing being jittered [*F*_(1, 9)_ = 0.45 *p* = 0.52], or of the length of the ISI [*F*_(3, 27)_ = 0.247, *p* = 0.76]. The interaction between timing jitter and ISI length [*F*_(3, 27)_ = 1.77, *p* = 0.18], between frequency jitter and ISI length [*F*_(3, 27)_ = 0.53, *p* = 0.67], and between timing jitter and frequency jitter [*F*_(3, 27)_ = 0.06, *p* = 0.82] were all not significant. The three-way interaction between timing jitter, frequency jitter and ISI length was also not significant [*F*_(3, 27)_ = 0.68, *p* = 0.53] (see Figure 3).

### Discussion

Experiment 2 employed two manipulations to increase difficulty of the objective within-stream intensity deviance detection task (employed to measure segregation thresholds). This was done to investigate whether the Δf thresholds in the manipulated conditions would then vary depending on the ISI of the tone sequence. Although one of the manipulations (base frequency jitter) was successful, task performance was still not influenced by ISI length. This leads back to the question of whether the lack of an increase in thresholds is really just a ceiling effect, or whether the task is dependent on other mechanisms as well.

One possible explanation for the lack of variation in the intensity task thresholds for different ISI values was that subjects might be capable of listening out for every third tone. This might allow them to continue solving the task when they can no longer segregate the streams (Dowling et al., 1987). If subjects adopted such a strategy, performance should worsen when, due to the removal of the regularity of the timing, accurate timing of the tones is no longer possible (Andreou et al., 2011; Rimmele et al., 2012). This might imply that the relative contribution of other factors that can account for the subject's performance, such as streaming ability, would increase. However, our results do not show a significant effect of timing jitter on the thresholds, which might suggest that this strategy was not used by the subjects. Alternatively, it could be that the amount of timing jitter was not sufficient to prevent subjects from focussing on every third tone. It is therefore not possible to determine conclusively why the Δf thresholds of the timing jitter condition do not vary depending on the ISI of the tone sequence.

Another possible explanation for the lack of variation in the intensity task thresholds for different ISI values would be that there was a transfer effect across trials. If the task was facilitated by the subjects being able to “hold on” to the lower tone from the previous trial, they might be able to solve the task at lower thresholds than would otherwise be expected. As transfer effects have been shown to be drastically reduced by noticeable parameter changes (Anstis and Saida, 1985; Cusack et al., 2004; Roberts et al., 2008; Haywood and Roberts, 2010), the jitter of the base frequency should be able to reduce any carry-over effect, lessening the probability of subjects experiencing streaming build-up or transfer across multiple sequences.

Indeed, the base frequency jitter condition, where the lower tone sequence frequency was changed after each trial, did significantly increase the thresholds when compared to the control condition, indicating that the difficulty of the task was increased by the jitter. This would suggest that there was a carry-over effect in the original intensity task of Experiment 1, which should at least be reduced in the jitter condition. However, in spite of the increase in thresholds, there was still no significant effect of ISI on the measured Δf thresholds within this condition. This would suggest that in spite of reducing the carry-over effect that was present in the original intensity task, the adapted base frequency jitter condition still does not allow the effective use of the intensity task for determining stream segregation thresholds using the staircase procedure. It therefore seems likely that performance in this task is influenced not only by the factors actively being manipulated (ISI and Δf as determinants of stream segregation). Instead, it would appear that performance is affected additionally or even exclusively by other factors, such as cognitive strategies for solving the task.

We would therefore suggest that if the intensity task is used to examine stream segregation in a staircase procedure, the base frequency should be altered after each step to avoid such carry-over effects. However, we would also question whether the intensity task is actually suitable for use in such a procedure.

## General discussion

The current study sought to examine auditory stream integration and segregation thresholds as measured using staircase versions of two tasks, a within-stream deviance detection task (facilitated by segregation) and an across-stream temporal judgment task (facilitated by integration). Combining these objective measures with subjective streaming judgments, using highly similar stimuli within one study, with the same subjects performing all tasks, allowed us to draw a direct comparison between the performances in the different tasks, as well as between task performance and subjective perception. This meant that it is possible to assess how effective a predictor the objective task was of the subjective perception. Further, by examining both, integration and segregation thresholds, a “map” can be drawn of the parameter space tested, describing whether it is possible to hear an integrated or a segregated percept at a particular point, or whether both percepts are possible. This was done efficiently, though some questions remain about the validity of the objective segregation task (based on intensity deviance detection) when combined with the staircase procedure.

A key benefit of the procedure described here is that by mapping ISI thresholds at fixed Δf levels and vice versa, a larger parameter space can be covered in a relatively short space of time, without making prior assumptions about the expected thresholds. For the study detailed in Experiment 1, each subject completed 12 objective tasks and 8 subjective threshold assessments, each taking between 1 and 5 min to run. This meant that including breaks, the entire session lasted about 60–80 min. This is a feasible length of time for within-subject comparisons. The tasks were chosen to hopefully make them transferable to special populations, such as children, cochlear-implant users, and patients with auditory disorders, who may not cope too well with longer or multiple sessions. Note that the enhanced efficiency would also be beneficial in other contexts: for instance, prior to neurophysiological measurements of streaming correlates, the relevant subset of the current staircase measurements can be selected for quickly determining optimal combinations of Δf and ISI.

There is, to our knowledge, only one study by Micheyl and Oxenham (2010) that previously examined both integration and segregation thresholds in the same subjects using subjective and objective measures. Our study significantly enhances the feasibility of such comparisons by demonstrating that a staircase procedure for the direct assessment of streaming thresholds can be used for this purpose as well. The previous study by Micheyl and Oxenham (2010) used timing deviations to measure stream integration thresholds. Apart from small procedural differences, their task was structurally very similar to the rhythm task used successfully in the current study to measure integration thresholds. A major difference between the two approaches is that Micheyl and Oxenham measured thresholds of a third, task-relevant variable at fixed points of ISI and Δf, while the current approach permits a direct measurement of the parameters determining the streaming percept (i.e., ISI or Δf). This direct measurement renders the measurement more time-efficient. The current results are generally consistent with those obtained by Micheyl and Oxenham (2010), with a clear interdependency of ISI and Δf in determining both subjective and objective measures of stream integration, and a clear correlation between these subjective and objective measures. This suggests that across-stream temporal judgments are a valid objective indicator of the subjective experience of an integrated percept, both in the version used by Micheyl and Oxenham and in the current staircase adaptation, and either task can be used depending on how many assumptions can be made in advance about the parameter space. One notable exception is the condition where subjective integration thresholds were measured with fixed Δf and variable ISI. This was the only condition in which the results did not correlate with the other subjective and objective integration threshold measurements. We therefore suggest that the self-adjustment of ISI values for approaching the subjective stream integration threshold needs further exploration and should not yet be applied in threshold measurements.

The current study employed an intensity deviance detection task to assess the segregation thresholds. It was not possible to demonstrate an effect of both Δf and ISI on these objective segregation threshold measurements, even though Micheyl and Oxenham were able to find such effects within the 7 subjects that completed all conditions in their study. The most likely reason for this discrepancy lies in the nature of the objective task. Note that Micheyl and Oxenham's within-stream timing task is more similar to the rhythm task of the current study, which was found to be reliable in the integration threshold measurements. It remains to be investigated whether this within-stream timing task would yield consistent results when combined with a staircase procedure for directly assessing streaming thresholds. The current within-stream deviance detection task proved to be less suitable for use in the procedure used here, even though in Experiment 1, this task was employed very similarly to how it has been used in the literature. However, it has been well-established in electrophysiological studies and, more recently, has also been employed in fMRI studies of auditory streaming (Sussman et al., 2001, 2007a; Winkler et al., 2003a,b; Sussman, 2005; Sussman and Steinschneider, 2006, 2009; Lepistö et al., 2009; Deike et al., 2010). The main difference in the current experiment to the usual method used with the intensity deviant was that the sequences were broken down into small pieces to allow the application of the staircase protocol, rather than presented as a continuous task. In this study's original version of the task, subjects did not show an effect of ISI on the Δf threshold required for successful task performance, contrary to what has been observed with other methods of measuring stream segregation (Denham et al., 2010, 2013; Micheyl and Oxenham, 2010).

Experiment 2 was set up to attempt to alleviate this problem by employing two modifications to the stimulus protocol. Although one of these modifications (changing the base frequency from trial to trial) was successful in increasing task difficulty, we still found no effect of ISI on the Δf threshold required to solve the task. It appears that task performance is not exclusively influenced by stream segregation but also by other, possibly more cognitive factors. The validity of this task as a pure measure of stream segregation thresholds when combined with a staircase procedure must therefore be questioned. Whether this limitation extends to the continuous version of the within-stream deviance detection task is beyond the scope of the current manuscript. In view of the present data, the circumstances under which this task is solvable by cognitive strategies, rather than by relying on stream segregation, should be investigated carefully.

Based on the present results, the rhythm task for objectively measuring stream integration thresholds lends itself much more readily to be used in combination with a staircase procedure for the direct assessment of streaming thresholds. We have shown here that this protocol yields threshold data with high internal consistency at the group level. We currently see the greatest benefit of the suggested method in quickly narrowing down the relevant parameter space for streaming assessments in groups with little prior knowledge as to where their streaming thresholds are expected to lie. Whether this protocol can be extended to streaming threshold measurements at the level of individual subjects must be carefully evaluated in future studies. These should include investigations of re-test reliability at the individual level, which was beyond the scope of the present study. Given the group-level consistency of the present data for the various versions of the rhythm task (tasks 2, 3, and 4 of Experiment 1), we would suggest that it is sufficient to use only one of the versions for assessing stream integration thresholds, which would lead to a further reduction in measurement time. In exchange, it may be necessary to collect more than one staircase threshold measurement for each pre-set level of one of the parameters in order to obtain reliable data at the individual level. This issue of a trade-off between measurement time and reliability at the single subject level must await further examination (see also Micheyl and Oxenham, 2010).

In conclusion, the present study shows that a staircase procedure can be combined successfully with across-stream temporal judgments as a classical tool for measuring stream integration. This combination allows a fast coverage of the parameter space, resulting in a quick assessment of streaming thresholds. This can be done without the burden of limiting the range of observations a priori by pre-determining the parameter space for the variables affecting the streaming percept. Future studies should disclose an objective task for measuring stream segregation that is equally suited for combination with the staircase procedure.

### Conflict of interest statement

The authors declare that the research was conducted in the absence of any commercial or financial relationships that could be construed as a potential conflict of interest.
